# Investigation of the *cis*–*trans* structures and isomerization of oligoprolines by using Raman spectroscopy and density functional theory calculations: solute–solvent interactions and effects of terminal positively charged amino acid residues†

**DOI:** 10.1039/d0ra05746k

**Published:** 2020-09-17

**Authors:** Mei-Chun Huang, Wei-Hao Chen, Chen-Wei Huang, Kuei-Yen Huang, Jia-Cherng Horng, Michitoshi Hayashi, I.-Chia Chen

**Affiliations:** Department of Chemistry, National Tsing Hua University 101, Sec. 2, Kuang-Fu Road Hsinchu Taiwan 30013 Republic of China icchen@mx.nthu.edu.tw; Center for Condensed Matter Sciences, National Taiwan University Taipei Taiwan 10617 Republic of China

## Abstract

Using low-wavenumber Raman spectroscopy in combination with theoretical calculations *via* solid-state density functional theory (DFT)-D3, we studied the vibrational structures and interaction with solvent of poly-l-proline and the oligoproline P12 series. The P12 series includes P12, the positively charged amino acid residue (arginine and lysine) N-terminus proline oligomers RP11 and KP11, and the C-terminus P11R and P11K. We assigned the spring-type phonon mode to 74–76 cm^−1^ bands for the PPI and PPII conformers and the carbonyl group ring-opening mode 122 cm^−1^ in the PPI conformer of poly-l-proline. Amide I and II were assigned based on the results of mode analysis for O, N, and C atom displacements. The broad band feature of the H-bond transverse mode in the Raman spectra indicates that the positively charged proline oligomers PPII form H-bonds with water in the solid phase, whereas P12 is relatively more hydrophobic. In propanol, the PPI conformer of the P12 series forms less H-bond network with the solvent. The PPII conformer exhibits a distinct Raman band at 310 cm^−1^, whereas the PPI has bands at 365, 660, and 960 cm^−1^ with reasonable intensity that can be used to quantitatively determine these two conformational forms. The 365 cm^−1^ mode comprising the motion of a C=O group turning to the helix axis was used to monitor the isomerization reaction PPI ↔ PPII. In pure propanol, RP11 and KP11 were found to have mostly PPI present, but P11R and P11K preferred PPII. After adding 20% water, the PPI in P11R and P11K was completely converted to PPII, whereas a small fraction of PPI remained in RP11 and KP11. The substituted positively charged amino acid affected the balance of the PPI/PPII population ratio.

## Abstract 2

The low-wavenumber Raman spectra in combination with theoretical calculations *via* solid-state density functional theory (DFT)-D3 are displayed. The vibrational structures and interaction with solvent of poly-l-proline and the oligoproline P12 series are identified.

## Graphical abstract

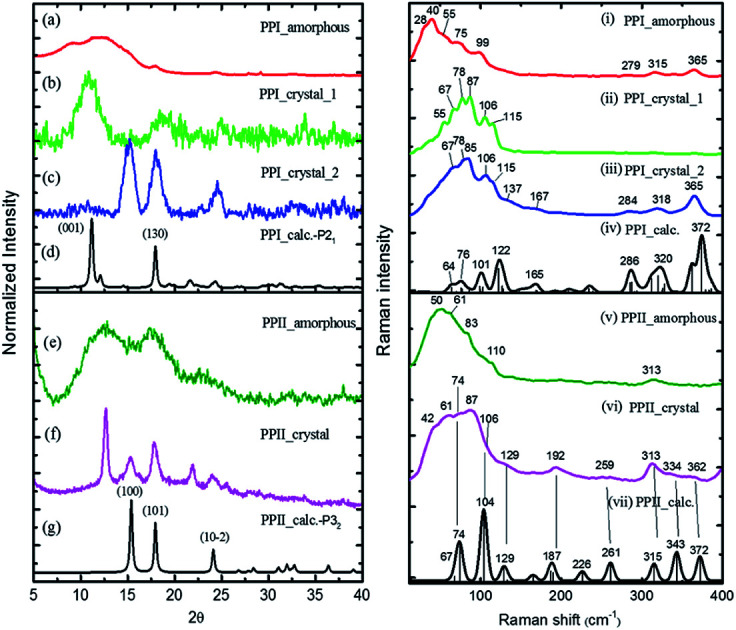

## 1. Introduction

**Scheme 1:**
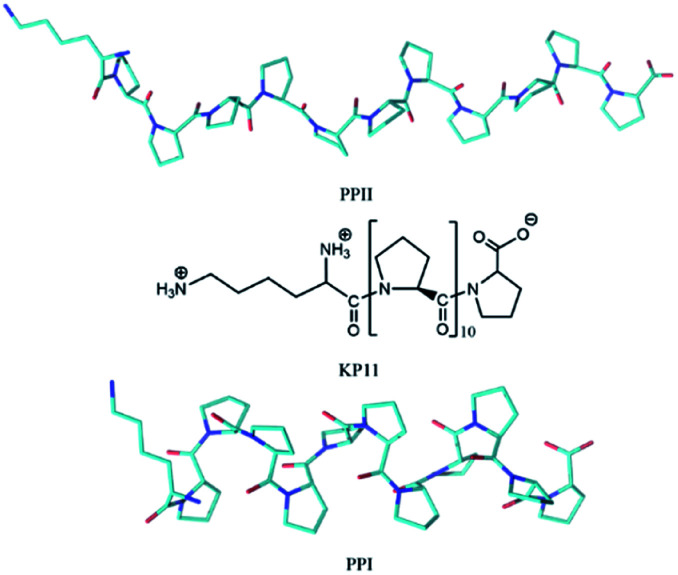
Structure of KP11 and its PPI and PPII conformations.

Proline is the only cyclic proteinogenic amino acid with a secondary amine forming the peptide bond, and the cyclic side chain provides proline with exceptional rigidity. Whereas most amino acids adopt *trans* peptide bonds, proline is able to form both *cis* and *trans* isomers because of the steric constraints on the conformation caused by the pyrrolidine ring and a lack of intramolecular hydrogen bonds.^1,2^ Oligoprolines convert between these two helical conformations depending on their environment. In aqueous solution, the polyproline II (PPII) *trans* conformation is favored due to the formation of hydrogen bonds (H-bonds) between the carbonyl groups on the backbone and water, whereas in hydrophobic solvent such as aliphatic alcohols, the polyproline I (PPI) *cis* conformation is preferred.^3^ The PPII *trans* and PPI *cis* structures of KP11 are depicted in Scheme 1. Proline *cis*–*trans* isomerization plays an important role in various biological processes, such as rate-determining steps of protein folding, autoinhibition control of a signal protein, and channel gating, *etc.*^4–8^

PPII is a dominant conformation not only in collagen but also in elements of the folded and unfolded proteins. The PPII helix is a more elongated left-handed helical structure with an axial repeat of 9.60 Å, composed of three prolyl residues per turn. The PPI helix is a right-handed helical structure with an axial repeat of 8.93 Å, composed of 3.3 prolyl residues per turn.^9–13^ Ruggiero *et al.* used terahertz spectroscopy to obtain the frequencies of phonon modes of poly-l-proline. Combining their results with calculations, they assigned the spring-type bands, consequently revealing the elasticity of poly-l-proline helices.^11^

Several factors influence the conformational stability of the polyproline helix. At low temperatures, the PPI helix is favored in less-polar solvents; as the temperature is increased, the PPII helix forms. In polar solvents, PPII is favored at all temperatures.^14–16^ Proline *cis*–*trans* isomerism also depends on either stereoelectronic or steric effects that restrict main-chain torsion angles.^17–21^ Peptides containing aromatic residues favor the *cis* conformation *via* both the hydrophobic effect and aromatic–proline interactions, C–H/π interactions.^22–25^ The PPI helix is stabilized relative to the PPII helix by positively charged functional groups at the N-terminus and negatively charged functional groups at the C-terminus.^26–28^ Shi *et al.* detected the ion mobility and employed mass spectrometry to examine the detailed intermediate steps associated with the process of oligomer proline-13 (P13) conversion from the PPI to the PPII helix. Collision cross section distributions of P13 [M + 2H]^2+^ ions obtained at different transition times indicated the presence of two major conformers, identified as the PPI and PPII helices, and six conformers that appeared as subpopulations of polyproline. They suggested that prolines sequentially flip from *cis* to *trans* starting from the N-terminus.^1,29,30^

Water plays a critical role in maintaining the conformation of collagen molecules and the mechanical properties of collagen fibrils. Water bridges are networks of water molecules that link nearby hydrogen bond acceptors and/or donors on proteins.^3,31–35^ The characteristic ring of polyproline precludes the nitrogen atom of the prolyl bond from engaging in hydrogen bonding. This feature not only affects the structure of the helices but also the interaction with solvents. The *cis*–*trans* conformations are linked to the orientation of the carbonyl groups C=O. In PPI, these groups are almost parallel to the axis of the compact helix and are shielded from the solvent by proline rings; in PPII, the C=O groups are mainly perpendicular to the axis of the helix, and because the helix is more elongated, the carboxyl oxygen is more exposed to solvents.^26,36^ This demonstrates how crucial water is for the stability of the PPII helix relative to that of the PPI helix.

In the present study, we used a low-wavenumber Raman spectroscopy technique to study the structure and intermolecular interactions of polyproline in solid. Combining Raman spectroscopy with theoretical calculation using density functional theory-D3 (DFT-D3) and considering the intermolecular interaction on the crystal structure, we were able to assign the phonon modes in the PPI and PPII configurations. Raman spectroscopy is complementary to infrared spectroscopy and has a superior signal-to-noise ratio in the low-wavenumber region. The *cis*–*trans* isomerization affected by the terminal positively charged residues was studied by Huang *et al.* using circular dichroism (CD), and they demonstrated that a positively charged residue at the C-terminus increases the stability of a PPII helix.^27^ Here, we studied the isomerization conversion of N- and C-substituted arginine oligoproline-11 (RP11, P11R) and lysine substituted P11 (KP11, P11K) using Raman spectroscopy. In addition, the low-wavenumber Raman spectra reveal the H-bond information in both solid crystalline packing and solutions.

## 2. Experimental

### Peptide synthesis and sample preparation

Sample oligoproline-12 (P12) was synthesized on an automated PS3 peptide synthesizer (Protein Technologies). A standard solid-phase method was used as described in detail previously.^27^ N- and C-substituted arginine oligoproline-11 (RP11, P11R) and lysine P11 (KP11 and P11K) (Kelowna International Scientific) were checked without emission then were used as received without further purification. PPII polyproline was recrystallized by dissolving poly-l-proline (Sigma, P2254, MW 1000–10 000, PPII) in a 1 : 3 formic acid to water mixture, incubating the solution for 4 d, and allowing the solution to evaporate. PPI was recrystallized in a 1 : 9 formic acid to 1-propanol (prOH) solution. After the peptides were incubated for more than 6 d, prOH solution was evaporated, and then ethanol was added and subsequently allowed to evaporate to obtain the crystals.

Pure deionized water (Milli-Q Millipore, 18.2 MΩ cm^−1^) and 1-propanol (prOH, HPLC grade) were used in all preparations. Samples in water were prepared at 24 mM. All peptides except P12 were dissolved in prOH at 33 mM and then incubated at 296 K for at least 4 d to ensure an equilibrium condition in which PPI was the dominant conformer. P12 was dissolved in 94% (v/v) prOH/water because of its solubility. The PPI → PPII conversion was initiated by dilution of the prOH solution with portions of H_2_O to ∼80% (v/v). Each solution was incubated at room temperature for 2 h to ensure the equilibrium distribution. Poly-l-proline was unable to dissolve in prOH; hence, the Raman spectra could not be obtained in solution for the polymers.

### Measurements

A Raman spectrometer with He–Ne laser as the excitation light source was used to record the spectra. The laser light traveled through a laser line filter and was focused onto a sample with an objective (10×, NA 0.26). The collimated scattered light was collected with the same objective and passed through two Bragg Gratings (BragGrate notch filters, OptiGrate), which rejected the scattered Rayleigh light. The scattered signal was dispersed by a monochromator (length, 0.5 m; grating, 600 grooves per mm) and detected by a charge-coupled device cooled with liquid nitrogen. All measurements were performed at room temperature (296 K). The band position was determined within 2 cm^−1^.

To obtain the low-wavenumber region quantitatively, the experimental Raman spectra were converted into the reduced Raman intensity by using the following equation:^37^1
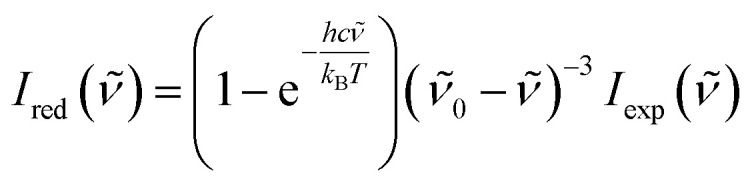
where *ν̃* is the wavenumber of Stokes–Raman shift, *ν̃*_0_ is the wavenumber of the excitation laser light, *h* is the Planck constant, *c* is the speed of light, *k*_B_ is the Boltzmann constant, and *T* is the absolute temperature.

X-ray powder diffraction measurements of the samples were performed using the Bruker D8 Advance. The patterns were obtained using CuKα_1_ (*λ* = 1.5406 Å) over the 2*θ* range of 5–40 degrees and collected using a tube voltage of 40 kV and current of 40 mA.

### Computation simulations

The geometry was optimized and the phonon frequencies of the solids were obtained using the software package CRYSTAL 17,^38^ and the initial input geometries of the 3D PPI and PPI helices were based on the structural data derived from X-ray diffraction.^11^ All calculations were performed using the B3LYP hybrid density functional^39–42^ and basis set 6-31G(d,p). The B3LYP-D3 dispersion approach, augmented by Grimme's dispersion force correction, was used to incorporate the London dispersion forces into the DFT functional.^43,44^ The basis set superposition error (BSSE) was corrected through the geometrical counterpoise (gCP) scheme in the optimizing steps.^45–47^ The full optimization including both the unit cell and the atomic coordinate in the cell was employed. The total energy convergence criteria for the geometry optimization and frequency calculations were set to 10^−8^ and 10^−10^ hartree, respectively. A shrinkage factor (4, 4) was used to define the commensurate grid and sampling rate of *k* points in the reciprocal space for solid-state calculations. Raman intensities were simulated at the B3LYP-D3/6-31G(d,p) level with the coupled-perturbed Kohn–Sham scheme.^48–50^ The optimized crystal volume deviates less than 0.5% from the experimental and the lattice sizes are listed in the Table S1.† The calculated frequencies of phonon modes are listed in Tables S2–S5.†

The Gaussian software package^51^ was used to obtain the geometries, dipole moment, and energies of both isolated and solvated molecules. The initial input geometries were generated with idealized torsion angles for the PPI and PPI helices based on the X-ray structures^11,52^ For PPI and PPII conformers of P12 in the gas phase, the optimizations were performed at the B3LYP/6-31G(d) level. In solution, the continuum model CPCM,^53,54^ which considers no direct intermolecular interactions such as hydrogen bonds between the solvent, was used. For each peptide, the optimizations and the vibrational wavenumbers were calculated at the B3LYP/CPCM (H_2_O or 1-propanol)/6-31G(d) level. Calculated vibrational frequencies usually overestimate the experimental fundamentals due to the inaccurate description of the electron–electron interaction and the neglect of anharmonicity. The scaling factors of 0.9603 for the B3LYP/6-31G(d) and 0.9608 for the B3LYP/6-31G(d,p) method in this work are commonly used to correct the calculated values to match the experimental vibrational frequencies. In the high-wavenumber region, we compared our experimental spectra with the results of GAUSSIAN calculations. To achieve accurate energy, the method and basis set M06-2X/def2-TZVP^55^ was used for the P12 series at their optimized geometries at B3LYP/6-31G(d).

## 3. Results and discussion

### Poly-l-proline

**Fig. 1:**
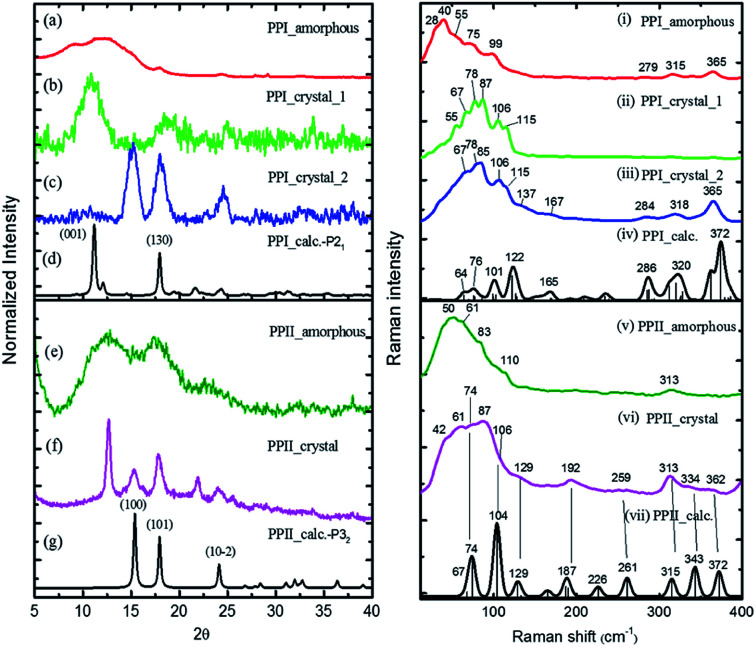
((a)–(g)) powder X-ray curves and calculated (black, (d), (g)) using PPI *P*2_1_ and PPII *P*3_2_ crystal data, and ((i)–(vii)) low-wavenumber Raman spectra of poly-l-proline I and II with simulated Raman spectra (black, (iv), (vii)) using CRYSTAL 17 at the B3LYP/6-31G(d,p) level with the coupled-perturbed Kohn–Sham scheme.

The powder X-ray diffraction curves and the Raman spectra of both PPI and PPII conformations of poly-l-proline are displayed in Fig. 1. The poly-l-proline was a cotton-like powder, type PPII, when purchased and was subsequently recrystallized. We obtained one kind of PPII solid having broad powder X-ray diffraction (PXRD) peaks centered at 2*θ* = 12.6, 17.5, and 21.9° (Fig. 1e). The corresponding Raman spectrum is shown in Fig. 1v. The second kind of solid had narrower PXRD peaks with positions similar to those above plus extra peaks at 15.3, 17.8, and 24.1°, as shown in Fig. 1f. In fact, these new 2*θ* positions agree with those calculated using the known crystal structure *P*3_2_ of the PPII unit cell, *a* = 6.643 Å and *c* = 9.6 Å (Fig. 1g). Hence, this batch of solid had at least two different lattice unit cells. Water was used in crystallization, and the PPII conformation tended to form intermolecular hydrogen bonds (H-bonds) with water, in addition to the intra- and inter-peptide H-bond (shown in the ESI†). The transverse acoustic phonon mode of water has a broad band peaking at 60 cm^−1^. Hence, the broad feature in the wavenumber region of 40–60 cm^−1^ of the Raman spectrum (Fig. 1v) of the PPII amorphous solid was possibly due to the water H-bonds. The second batch of solid had less water content than the first batch (Fig. 1v) and a more distinct intramolecular phonon band structure *ca.* >60 cm^−1^ (Fig. 1vi).

**Fig. 2:**
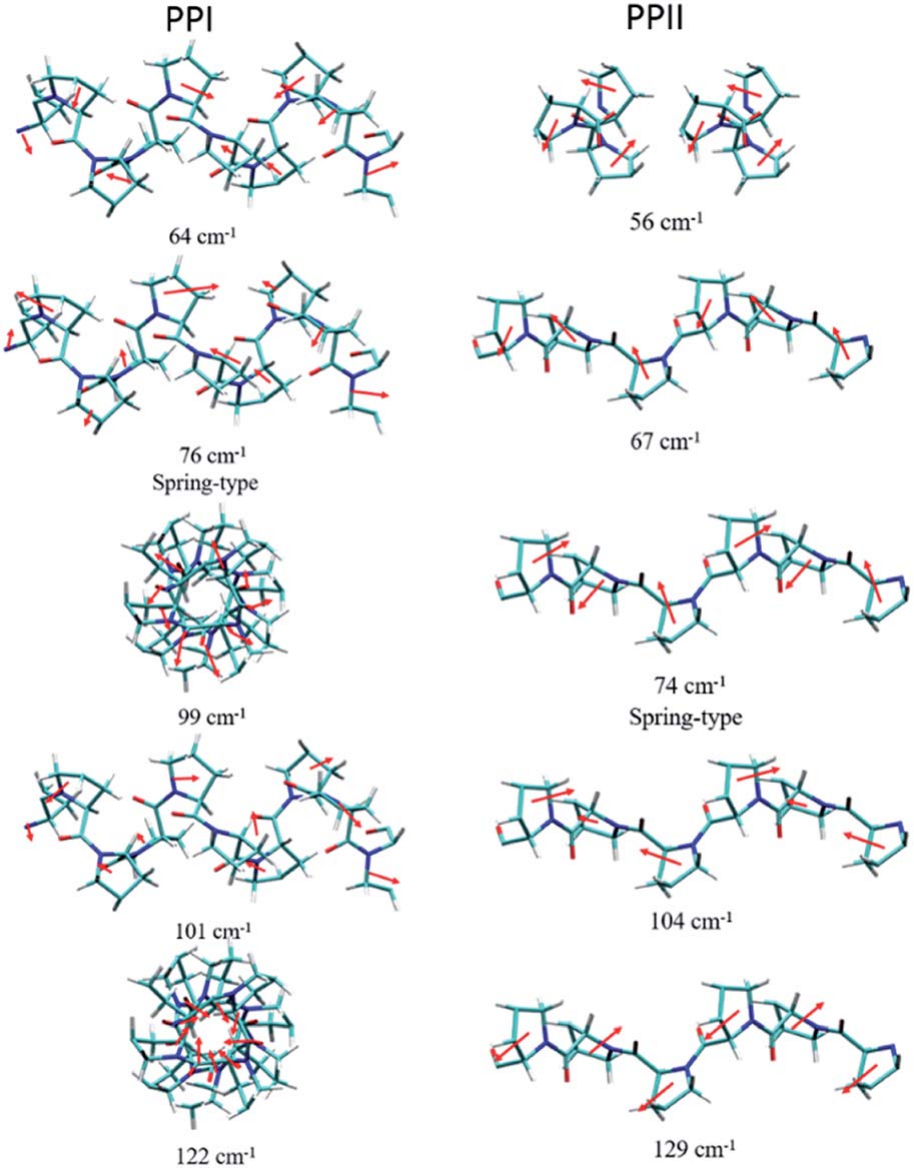
Calculated displacements of the atoms of phonon modes for poly-l-proline PPI (left) and PPII (right).

For assigning the phonon modes of polyproline, the CRYSTAL 17 package was used, and the calculated Raman spectra, based on the X-ray crystal data with the PPI space group *P*2_1_ and PPII *P*3_2_,^11^ are presented in Fig. 1iv and vii, respectively. For PPII, the calculated 56 cm^−1^ band was a libration mode and had a very weak intensity in the calculated curve which is not shown in Fig. 1vii. The other bands, 67, 74, 104, and 129 cm^−1^, were mainly attributed to proline backbone skeleton motion—the intramolecular vibrational modes and their motions are depicted in Fig. 2. These phonon modes exhibited the twisting motion of pyrrolidine rings in combination with CO bending, particularly the 74 cm^−1^ mode, which had a scissoring motion between alternative pyrrolidine rings manifesting a spring-type helix elongation/compression mode. Because of the low symmetry in polyproline, we expected all intramolecular phonon modes to appear in both infrared and Raman spectra despite of their intensities. Raman spectra have lower background signals; hence, these low wavenumber modes were better resolved. Our observations agree with the terahertz bands observed by Ruggiero *et al.*,^11^ which were approximately at 73, 98, and 130 cm^−1^ for PPII. Unlike us, they assigned the 98 cm^−1^ mode (cal. 100 cm^−1^) to spring-type motion. This mode corresponds to our calculated 104 cm^−1^ mode, as shown in Fig. 2 and is a pyrrolidine rings swing motion. Comparing these two motions, the 74 cm^−1^ mode exhibited more coil elongation/compression and thus was attributed to the spring motion. From the observed spectra, though the resolution was not satisfactory, we tentatively assigned the observed bands at 83 cm^−1^ in Fig. 1v and 74/87 cm^−1^ in Fig. 1vi to the spring-type 74 cm^−1^ (cal.) mode and the observed 110/106 cm^−1^ band to the 104 cm^−1^ (cal.) mode. The broad feature in the range of 40–110 cm^−1^ can be resulted from interaction by nearby co-solvents or adjacent polyprolines in the amorphous solid,^56,57^ which were not included in the calculations. The assignments on amide I and II were based on the results of mode analysis for O, N, and C atoms displacements to be 1650, 1424 cm^−1^ (1717, 1451 cm^−1^ cal.), respectively. Similar positions were assigned for PPI and these mode analyses are shown in Fig. S9 and S10.† All calculated modes are listed in the ESI Tables S2 and S3.†

The polyproline PPII powder was first converted to PPI, after which the structure was confirmed from the high-wavenumber region of Raman spectra. The PPI was then crystalized. One kind of solids obtained had an amorphous structure (Fig. 1a), and its Raman curve (Fig. 1i) showed more intensity in the low wavenumber region *ca.* <60 cm^−1^. These low wavenumber bands were attributed to the amorphous structure. The second batch of solids had broad PXRD peaks at 2*θ* = 11.2 and 18° (Fig. 1b), in agreement with the calculated positions based on space group *P*2_1_ for PPI (Fig. 1d). The corresponding Raman bands were at 55, 67, 78, 87, 106 and 115 cm^−1^ (Fig. 1ii). The third batch of crystals had narrower PXRD linewidths (Fig. 1c), but the 2*θ* positions were similar to those of PPII (Fig. 1g), with lattice symmetry *P*3_2_ and unit cell sizes. However, this batch exhibited PPI Raman spectra (Fig. 1iii) and low-wavenumber bands 67, 78, 85, 106, 115 cm^−1^.

According to the solid-state DFT calculation, these phonon bands *ca.* >100 cm^−1^ were mostly intramolecular vibrational modes. Slightly varied band features and positions in the low wavenumber range *ca.* <100 cm^−1^ resulted from varied intermolecular interactions and varied molecular packing in solid. For PPI, we obtained the calculated phonon modes, based on the known crystal structure, at 64, 76, 99, 101, and 122 cm^−1^, as shown in Fig. 1iv. Their atomic displacements, as displayed in Fig. 2, consisted of twisting of the pyrrolidine ring pairs for the first two modes, and then three later modes: scissoring of C=O groups, twisting of pyrrolidine rings, and bending of C=O from parallel to the helix axis to the perpendicular direction. From these twisting motions, the 76 cm^−1^ mode showed a spring elongation/compression motion; this assignment yielded a similar helix elasticity to the PPII configuration. The 122 cm^−1^ phonon motion mainly involved the C=O moving from parallel to perpendicular to the helix axis. Viewed along the helix line, this motion formed a helix circle-opening-like motion, as shown in Fig. 2. Isomerization of polyproline from the *cis*- to the *trans*-form required turning the carbonyl group to the position perpendicular to the helix line. Though this isomerization in oligomer proline is a sequential process, as suggested by Shi *et al.*,^29,30^ this mode ought to couple with the individual isomerization processes.

In the measured Raman spectra, the lack of the transverse H-bond mode feature at around 50 cm^−1^ indicated that the PPI poly-l-proline was more hydrophobic. The terahertz bands^11^ are around 66, 76, 99, and 125 cm^−1^, corresponding to our observed bands at 67, 78/87, 106/115, and 133 cm^−1^. Tentatively, they were assigned to the calculated modes 64, 76, 99/101, and 122 cm^−1^, respectively.

### P12 series

**Fig. 3:**
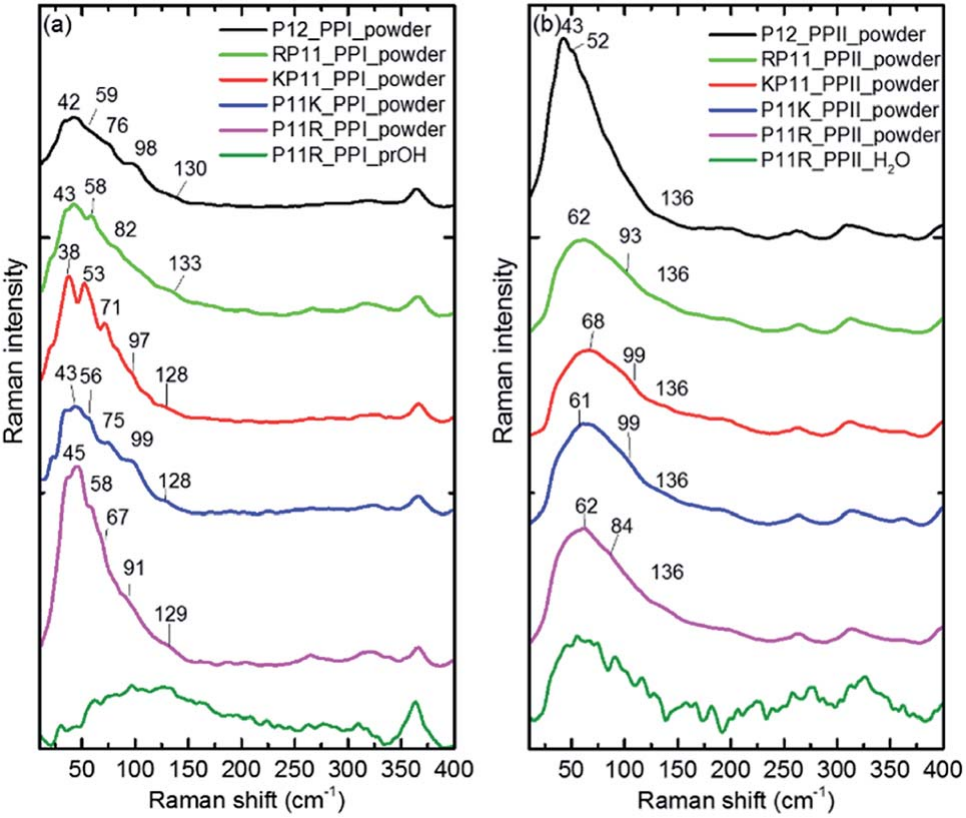
Raman curves of P12 series powder and P11R in propanol (PPI) and in aqueous solution (PPII), respectively for (a) PPI and (b) PPII. The intensity is normalized to the 365 and 310 cm^−1^ bands for PPI and PPII, respectively for the powder samples.

**Fig. 4:**
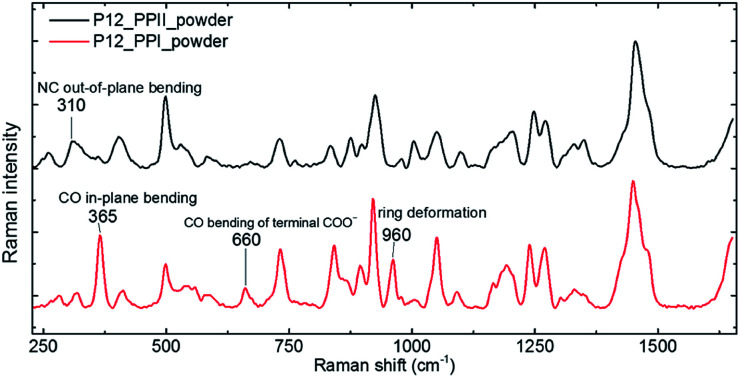
Raman spectra of P12 PPI (lower) and PPII (top) powders.

The P12 series contained oligomer P12, N-terminus substituted RP11 and KP11, and C-terminus P11R and P11K. Their Raman spectra in the low-wavenumber region of PPI and PPII powder are presented in Fig. 3. The other regions of Raman spectra are provided in the ESI† (Fig. S4 and S5†). With one amino acid substitution, some very minor changes in the amide bands were observed, but the overall spectra resembled because the oligomers consisted of mostly prolines and because R and K are not strong Raman scatters. However, the PPI and PPII conformers exhibited some distinct variations; for example, the PPII had a band at 310 cm^−1^, which was assigned to N–C out-of-plane bending. Instead, PPI had a sharp band at 365 cm^−1^, which was assigned to CO in-plane (pyrrolidine ring plane) bending (CO bending out of helix axis), as depicted in Fig. 4 for P12. Moreover, PPI had extra sharp bands at 660 and 960 cm^−1^, which were assigned to CO bending of the terminal COO^−^ and to pyrrolidine ring in-plane deformation, respectively. These two bands showed no intensity in the PPII samples. Similar variations were found for the substituted oligomers. These vibrational bands were also suggested by Rippon *et al.*^58^ to be used to distinguish the two forms. These variations provide a suitable means to differentiate and to quantitatively determine these two isomers.

The low-wavenumber Raman spectra of PPI powder of the P12 series had a broad band peaking near 45 cm^−1^ with a vague structure. The vague structure, marked in Fig. 3a, had peak positions at 38–45, 53–59, 67–82, 91–99, and 128–133 cm^−1^. The low wavenumber bands of <60 cm^−1^ had features similar to those of the PPI amorphous solid of poly-l-proline, indicating an amorphous structure of these solid samples having low-wavenumber acoustic phonon bands. The bands of >60 cm^−1^ were related to peptide skeleton motion involving twisting of the pyrrolidine ring pairs and C=O bending; hence, the mono-amino acid substitution had a limited effect on these mode positions. In solution, the P12 series PPI displayed similar Raman curves with a broad band centered at 100 cm^−1^ after subtraction of the pure prOH signal, and only the Raman spectrum of P11R in propanol is represented in Fig. 3a. This band position was different from the H-bond transverse mode of prOH. The peptide skeleton modes can be affected *via* solvent–solute and solute–solute interaction.^56,57^ Hence, this broad feature can be because of interactions of propanol and nearby oligoprolines to these low wavenumber motions.

For PPII, the Raman curve of the P12 powder displayed a broad band peaking at 43 cm^−1^ and a lack of a fine structure for individual phonon modes, indicating an amorphous structure in solid. However, the substituted oligomers had a broad band peaking at *ca.* 60 cm^−1^, implying the formation of H-bonds with water in solid phase. These data imply that P12 in solid is relatively more hydrophobic than the substituted P12. This can be understood due to the substituted oligoprolines being more hydrophilic because of an extra charge in amino acid K/R. The Raman curves of the P12 PPII series in aqueous solution were similar. Here, only the P11R curve is shown in Fig. 3b. These curves, with the pure water signal subtracted, showed similar broad features centered around 60 cm^−1^, resulting from H-bonding with water.

### Conversion PPI to PPII

Huang *et al.*^27^ used the circular dichroism (CD) technique and found that in *ca.* 95% propanol solution, P11R and P11K are already mainly present in the PPII configuration, while RP11, KP11 and P12 are mostly in PPI, and that as the concentration of *n*-propanol decreases, the signal peak of PPI gradually decreases. Below 50%, the PPII configuration is mainly present for all the P12 series. The CD spectra indicate the secondary structure of the proline chain, whereas the 365 cm^−1^ Raman band involves C–O bending out of the helix and only appears in PPI. The PPII configuration with a perpendicular CO bond shows no intensity. The peptide backbone is relatively rigid so this band is suitable to use to quantitatively determine the molecular configuration.

**Fig. 5:**
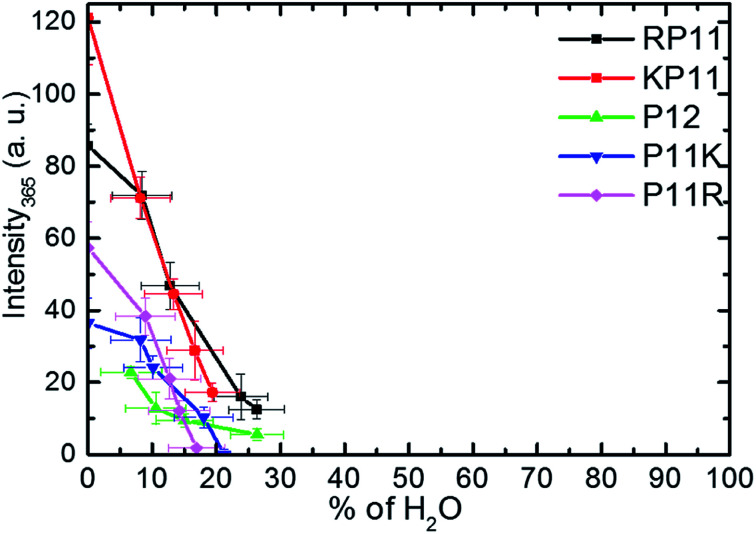
Plots of Raman intensity (±1*σ*) of the 365 cm^−1^ band *vs.* the percentages of water in prOH for P12 and its derivative charged peptides.

For the conversion measurements, first, the PPI sample of RP11, KP11, P11R, and P11K was dissolved in nearly 100% propanol. P12 was dissolved in 94% propanol solution at the same concentration. The Raman scattering intensity of the 365 cm^−1^ band was measured about 2 h after each addition of water aliquot and normalized to the prOH band intensity and concentration. The 365 cm^−1^ band intensity was found to decrease with the amount of water added, as shown in Fig. 5. When the oligoprolines were dissolved in pure prOH, we found that PPI in RP11 and KP11 was the major conformer and that the amount was greater than those of P11R and P11K. P12 was immersed in 94% prOH solution; hence, the proportion of the PPII type was greater. When water was added to the solution, the amount of PPI decreased sharply. At a concentration of about 20%, RP11 and KP11 retained a small portion of PPI, but P11R and P11K were only present as the PPII configuration. When the N-terminus was attached to a positively charged amino acid, the proline chain structure preferred the PPI in pure prOH over the C terminal substituted. The substituted positively charged amino acid affected the balance of the PPI/PPII ratio. These results agree with those from the CD measurements.

**Fig. 6:**
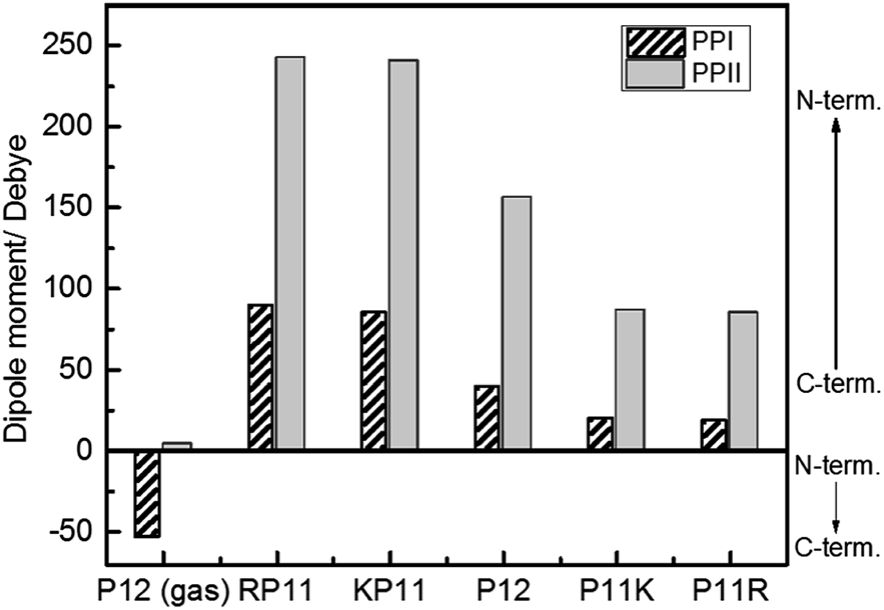
Calculated dipole moments of P12 series for the PPI and PPI conformations at the M06-2X/def2-TZVP//B3LYP/6-31G(d) level.

We used the Gaussian package to calculate the geometries and energies of the proline chains and to explain the relative stabilities of PPI and PPII. The input structure for the calculations of the PPII configuration of 7-mers was the crystal structure published by Wennemers *et al.,*^26^ and the PPI was from X-ray experimental data.^11^ After optimization, the geometries were added to form the 12-mers. Further optimization was performed to obtain the final geometries (Fig. S2†). The method of structural optimization and the basis set were B3LYP/CPCM (H_2_O or 1-propanol)/6-31G(d). The calculated results of the dipole moments for PPI in prOH and PPII in water at M06-2X/def2-TZVP//B3LYP/CPCM/B3LYP/6-31G(d) are presented in Fig. 6.

Because P12 in gas phase had no charges on the termini, the dipole moments were relatively low for PPII, 4.2 debye, and for PPI, −53 debye, in which the helical dipole moment was mainly provided by the CO groups. In solution, the charges at both ends were farther away in PPII than in PPI, so the terminal charge of PPII produced a greater dipole than that of PPI. Adding positively-charged arginine (Arg, R) at the N-terminus NH_3_^+^ with the negative charge C-terminus of proline increased the amount of charge and the dipole moment of RP11. The same was applied to KP11. In contrast, adding R on the C-terminus decreased the charge and consequently the dipole moment of P11R. Conclusively, the terminal charge size and dipole moments followed the order of RP11, KP11 > P12 > P11R, P11K.

The energy calculations were performed with the global hybrid functional with the 54% HF exchange method and superior basis set M06-2X/def2-TZVP to achieve more accurate energies. The calculated energy differences Δ*E* = *E*_PPI_ − *E*_PPII_ in water and in prOH, respectively and *E*_PPII_(H_2_O) − *E*_PPI_(prOH) are listed in Table 1. These energy differences were calculated for 7-mers and 12-mers, and the two displayed similar trends. The energy stability caused by H-bonding with the solvent was not considered here. Based on our Raman data, PPII formed H-bonding in aqueous solution, so the calculated value of *E*_PPII_(H_2_O) was overestimated, and Δ*E*(H_2_O) was underestimated. Nevertheless, without considering the H-bond stabilization, the values of Δ*E*(H_2_O) and Δ*E*(prOH) were all negative, indicating that PPI was more stable than PPII because the charge–dipole interactions in PPI occurred at shorter distances, hence, stronger interactions better stability. In both solvents, the order of Δ*E* values was RP11, KP11 < P11K, P11R because the high positively charged N-terminus further increased the charge–dipole interaction, leading to the lower energy of PPI.

**Table 1:** Energy differences (in kJ mol^−1^) between the PPI and PPII (Δ*E* = *E*_PPI_ − *E*_PPII_) conformations and for 7-mers and 12-mers in implicit water and prOH at the M06-2X/def2-TZVP//B3LYP/6-31G(d) level. Stabilization *via* H-bond in PPII is not included in the calculation

7-mer				12-mer			
Sample	Δ*E*(H_2_O)	Δ*E*(prOH)	*E*_PPII_(H_2_O) − *E*_PPI_(prOH)^a^	Sample	Δ*E*(H_2_O)	Δ*E*(prOH)	*E*_PPII_(H_2_O) − *E*_PPI_(prOH)^a^
P6R	−21.1	−25.5	6.1	P11R	−29.7	−36.4	12.9
P6K	−20.2	−24.1	2.5	P11K	−29.8	−37.0	11.5
				P12	−27.5	−39.7	17.9
KP6	−55.5	−69.4	38.5	KP11	−52.8	−70.4	32.9
RP6	−51.5	−65.5	35.9	RP11	−54.5	−73.1	36.6

a Energy difference of PPII in water and PPI in prOH.

A more negative value of Δ*E* meant that the PPI helix was more stabilized relative to the PPII helix, so the equilibrium shifted towards PPI. The value of Δ*E*(prOH) of RP11(KP11) was −73.1(−70.4) kJ mol^−1^ and that of P11R(P11K) was −36.4(−37.0) kJ mol^−1^. Hence, adding a positively charged amino acid to the N-terminus stabilized PPI. In 100% prOH, less H-bonding was found between solute and solvent; hence, from the energy difference, RP11(KP11) should have a larger fraction of the PPI than of P11R(P11K). If H-bonding stabilization was considered, the corrected value of Δ*E*(H_2_O) should become greater such that adding water shifted the equilibrium toward PPII. As listed in Table 1, the calculated value of the energy difference of PPII in water and PPI in prOH (*E*_PPII_(H_2_O) − *E*_PPI_(prOH)) of P11R was 12.9 kJ mol^−1^ and that of RP11 was 36.6 kJ mol^−1^. In P11R(P11K) a small energy difference was obtained. Experimentally, all PPI was converted to PPII when water was added up to 20% in P11R(P11K); hence, this reaction energy for PPI (prOH) → PPII (H_2_O) must become negative in this condition. When the corrected value of *E*_PPII_(H_2_O) is applied, the small *E*_PPII_(H_2_O) − *E*_PPI_(prOH) can become negative. However, in RP11 and KP11, this energy difference is greater (cal. 32.9 and 36.6 kJ mol^−1^), hence, the isomerization reaction shifted to PPII when adding water, but a small fraction of PPI remained.

## 4. Conclusions

Our results show that the intensity of the Raman 365 cm^−1^ band provides a convenient means to identify the *trans* and *cis* conformers of polyprolines and to study the isomerization reaction. The positively charged amino acid at the N-terminus or the C-terminus affects the dipole moment of the macro helix, the relative structural stability of PPI/PPII, and affinity to water. Consistent with the conclusion drawn from the CD measurements, the Raman bands show that the PPI helix is stabilized relative to the PPII helix by positively charged functional groups at the N-terminus. Our DFT results on dipole moments and energies provided the explanation on this phenomenon.

In the low-wavenumber region, the existence of a H-bond phonon band provides a method to identify the solvent H-bonding to the proline. The same technique can be applied to other polypeptides. The charged amino acids on the N- and C-termini of oligoprolines lead to hydrophilic PPII even in solid phase. In the P12 series, the PPI configuration is hydrophobic and forms no H-bonds in solid or with solvent prOH. For both configurations they can form varied crystal structures. The low wavenumber phonon bands < 100 cm^−1^, their positions and features are varied slightly from varied molecular packing in solid. We assign the spring-type mode involving the twist of pyrrolidine ring pairs and compression/elongation of peptide bonds to the 74–76 cm^−1^ band for both conformers based on both experimental and solid-state DFT calculation data.

## Conflicts of interest

There are no conflicts to declare.

## Supplementary Material

RA-010-D0RA05746K-s001

RA-010-D0RA05746K-s002

RA-010-D0RA05746K-s003
