# Supplementary material for: Investigation of the cis–trans structures and isomerization of oligoprolines by using Raman spectroscopy and density functional theory calculations: solute–solvent interactions and effects of terminal positively charged amino acid residues
Source: RSC Adv. 2020 Sep 17;10(57):34493–500. doi: 10.1039/d0ra05746k (PMC9056779; doi:10.1039/d0ra05746k)
Supplement: RA-010-D0RA05746K-s001 [file RA-010-D0RA05746K-s001.pdf]

Electronic Supplementary Material (ESI) for RSC Advances.
This journal is © The Royal Society of Chemistry 2020

Investigation of the *cis-trans* structures and isomerization of oligoprolines by using Raman spectroscopy and density functional theory calculations: solute-solvent interactions and effects of terminal positively charged amino acid residues

Mei-Chun Huang,[1] Wei-Hao Chen,[1] Chen-Wei Huang,[1] Kuei-Yen Huang,[1] Jia-Cherng Horng,[1] Michitoshi Hayashi,[2*] and I-Chia Chen[1]**

[1]Department of Chemistry, National Tsing Hua University, 101, Sec. 2, Kuang-Fu Road, Hsinchu, Taiwan 30013, Republic of China.

[2]Center for Condensed Matter Sciences, National Taiwan University, Taipei, Taiwan 10617, Republic of China.

Supporting Information: Raman spectra of P12 series and poly-L-proline, vibrational assignments, calculated dipole moments and electrostatic potential surfaces of P12 series, mode analysis on the amide-type motions, and H-bonds of poly- L-proline.

Table of Contents

1. Experimental and calculated Raman spectra of poly-L-proline

2. Dipole moments and electrostatic potential surfaces of peptides calculated using Gaussian package

3. Raman spectra of P12, P11X and XP11 in solid state and in solution

4. Assignments of vibrational modes to the observed Raman shifts

5. Computational simulated PPI and PPII crystal structures. ppi-optimized.cif, ppii-optimized.cif

Captions of figures and tables

Figure S1 Experimental (top) and calculated (below) Raman spectra of polyproline PPII crystals. The calculated curve is obtained by using Crystal 17 B3LYP-D3/6-31G(d). The assignments of vibrational modes are displayed.

Figure S2 Optimized geometry, dipoles, and electrostatic potential surfaces of P12, KP11, RP11, P11K, and P11R type PPI in propanol and PPII in H_2O using GAUSSIAN package.

Figure S3 Raman curves of poly-L-proline II and I and the calculated spectra using CRYSTAL 17 at the B3LYP-D3/6-31G(d,p) level.

Figure S4 Raman curves of P12 series (a) PPI in prOH and (b) PPII in aqueous solution. The intensity is normalized to the 365 and 310 cm^{-1} band, respectively.

Figure S5 Raman spectra of powder P12 series PPII form.

Figure S6 Raman spectra of the PPII form of P12 series in aqueous solution.

Figure S7 Raman spectra of the PPI form of P12 series powder.

Figure S8 Plots of circular dichroism (CD) signals at 214 nm (PPI) versus the percentages of prOH in phosphate buffer for P12 series peptides.

Figure S9 Mode analysis results of PPII conformation of poly-L-proline. Panels A and B show mode analyses on the amide-type motions and the collective type motions where all atoms participate in the motions, respectively. In the amide-type motions, the number of each type (O, N, C, and H) atom is counted if the displacement is larger than 0.01 Å for each direction (a, b, and c-axis). In the collective type motions, we use the total displacement larger than 0.059 Å which is determined on the basis of the three acoustic

phonon modes (ω=0). In Panel A, * represents the motions due to the ring deformations.

Figure S10 Mode analysis results of PPI conformation of poly-L-proline. Panels A and B show mode analysis results on the amide-type motions and the collective type motions in which all atoms participate in the motions, respectively. In the amide-type motions, the number of each type (O, N, C, and H) atom is counted if the displacement is larger than 0.01 Å for each direction (a, b, and c-axis). In the collective type motions, we use the total displacement larger than 0.032 Å which is determined on the basis of the three acoustic phonon modes (ω=0). In Panel A, * represents the motions due to the ring deformations.

Figure S11 (a) 1D and 3D crystal structure of poly proline peptide, (b) H-bond in polyproline II, and (c) H-bond in polyproline I.

Table S1. Experimental and simulated crystal lattice of PPI and PPII conformations of poly-L-proline by using Crystal 17 at the B3LYP-D3/6-31G(d,p) level with the coupled-perturbed Kohn-Sham scheme.

Table S2 Vibrational modes of poly-L-proline II and assignments based on CRYSTAL 17 calculations.

Table S3 Vibrational modes of poly-L-proline I and assignments based on CRYSTAL 17 calculations.

Table S4 Vibrational mode of the PPII form of P12 and their assignments based on Gaussian 09 calculations.

Table S4 Vibrational mode of the PPI form of P12 and their assignments based on Gaussian 09 calculations.

1. Experimental and calculated Raman spectra of poly-L-proline

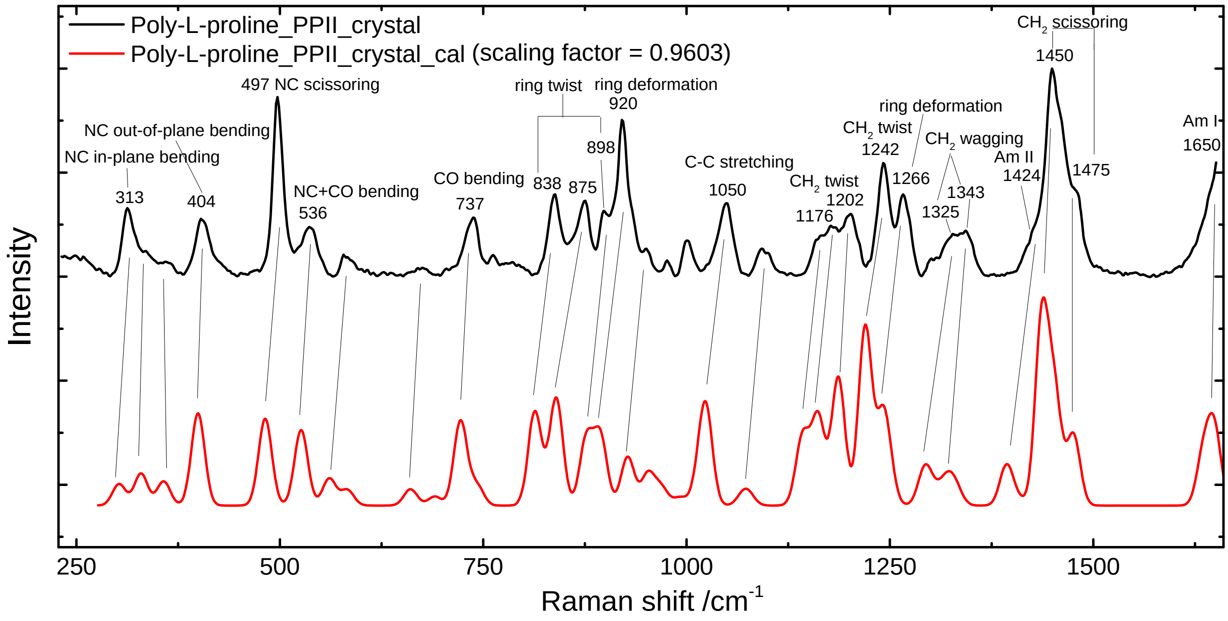

Figure S1. Experimental (top) and calculated (below) Raman spectra of poly-L-proline PPII crystals. The calculated curve is obtained by using Crystal 17 B3LYP-D3/6-31G(d). The assignments of vibrational modes are displayed.

2. Dipole moments and electrostatic potential surfaces of peptides calculated using Gaussian package

P12, PPI (gas)

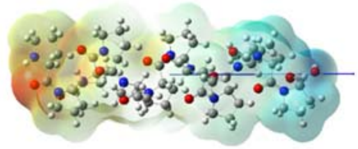

P12, PPII (gas)

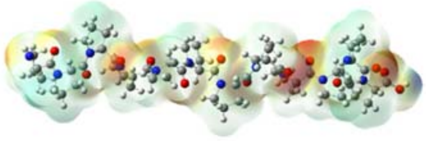

P12, PPI (prOH)

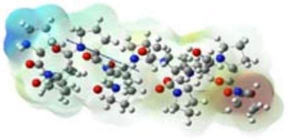

P12, PPII (H_2O)

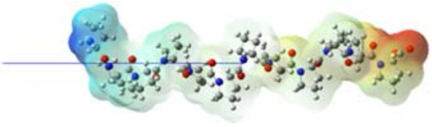

RP11, PPI (prOH)

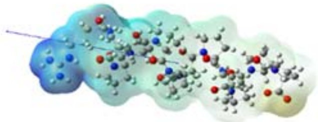

RP11, PPII (H_2O)

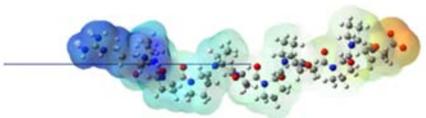

P11R, PPI (prOH)

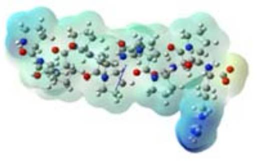

P11R, PPII (H_2O)

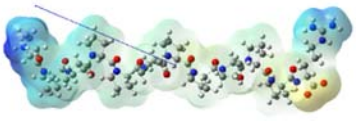

KP11, PPI (prOH)

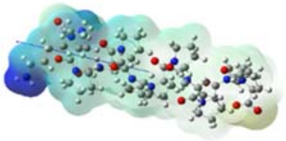

KP11, PPII (H_2O)

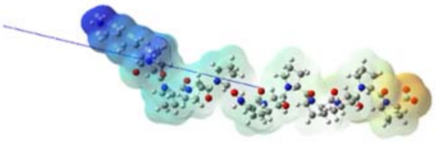

P11K, PPI (prOH)

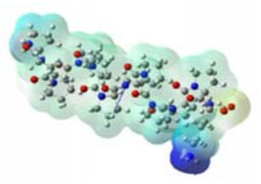

P11K, PPII (H_2O)

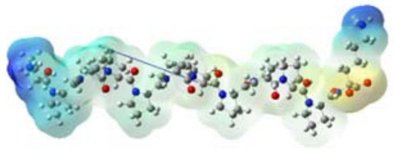

Figure S2. Optimized geometry, dipoles, and electrostatic potential surfaces of P12, KP11, RP11, P11K, and P11R type PPI in propanol and PPII in H_2O using GAUSSIAN package. N terminal is NH and C is COOH in gas phase and NH_2^+ and COO^- in water and 1-propanol for P12, P11K, and P11R and NH_3^+ and COO^- for KP11 and RP11.

3. Raman spectra of poly-L-proline, P12, P11X, and XP11 in solid state and in solution

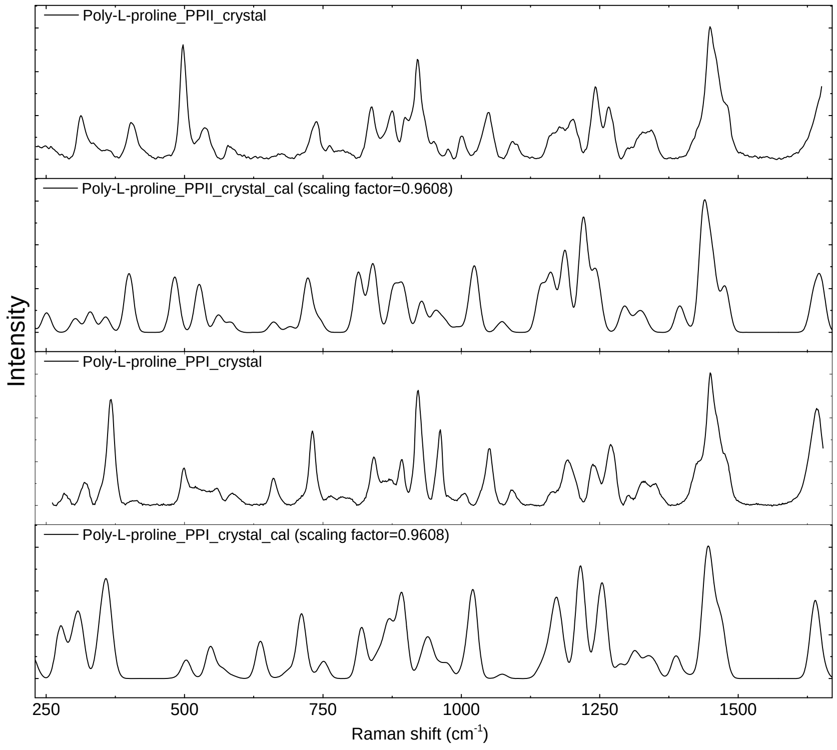

Figure S3. Raman curves of poly-L-proline II and I powder and the calculated spectra using CRYSTAL 17 at the B3LYP-D3/6-31G(d,p) level with the coupled-perturbed Kohn-Sham scheme.

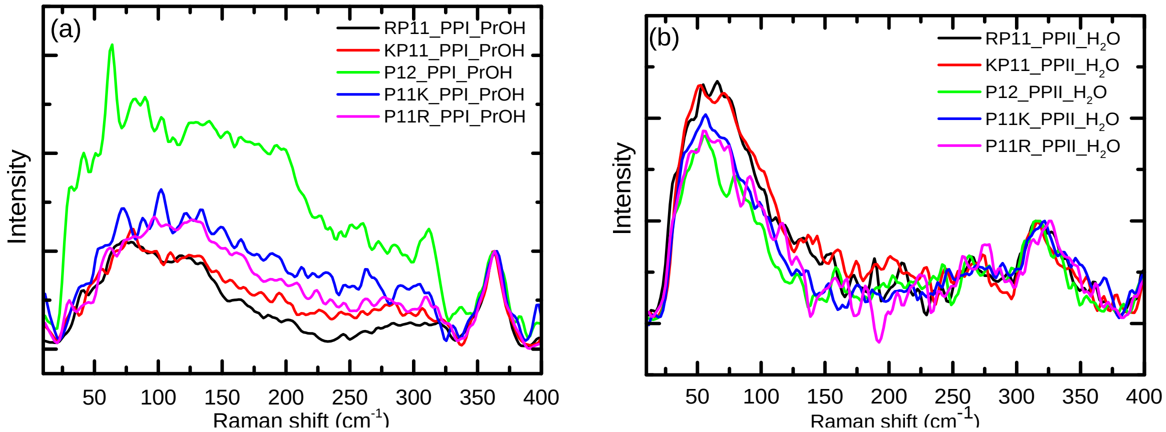

Figure S4. Raman curves of P12 series (a) PPI in prOH and (b) PPII in aqueous solution. The intensity is normalized to the 365 and 310 cm^{-1} band, respectively.

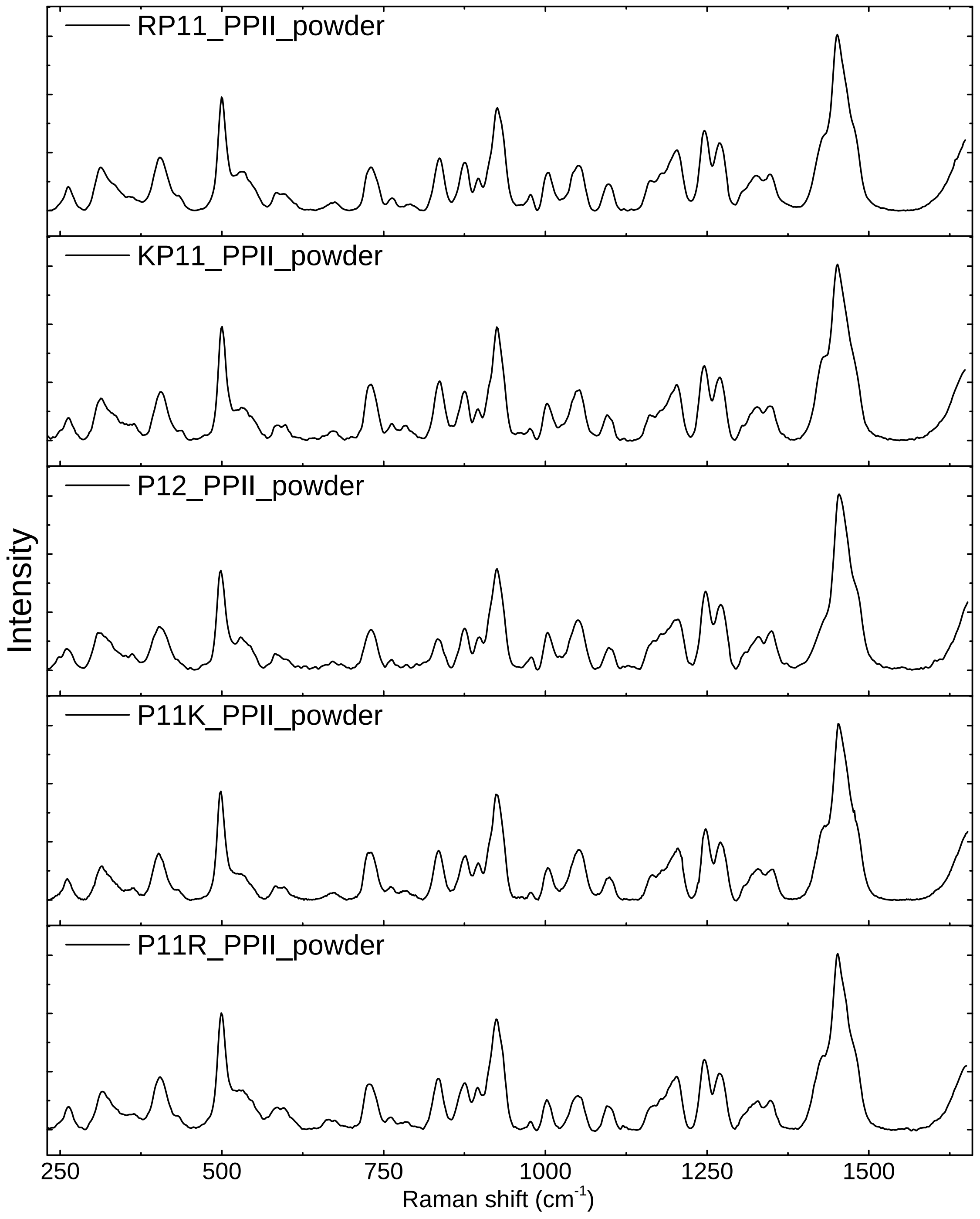

Figure S5. Raman spectra of powder P12 series PPII form.

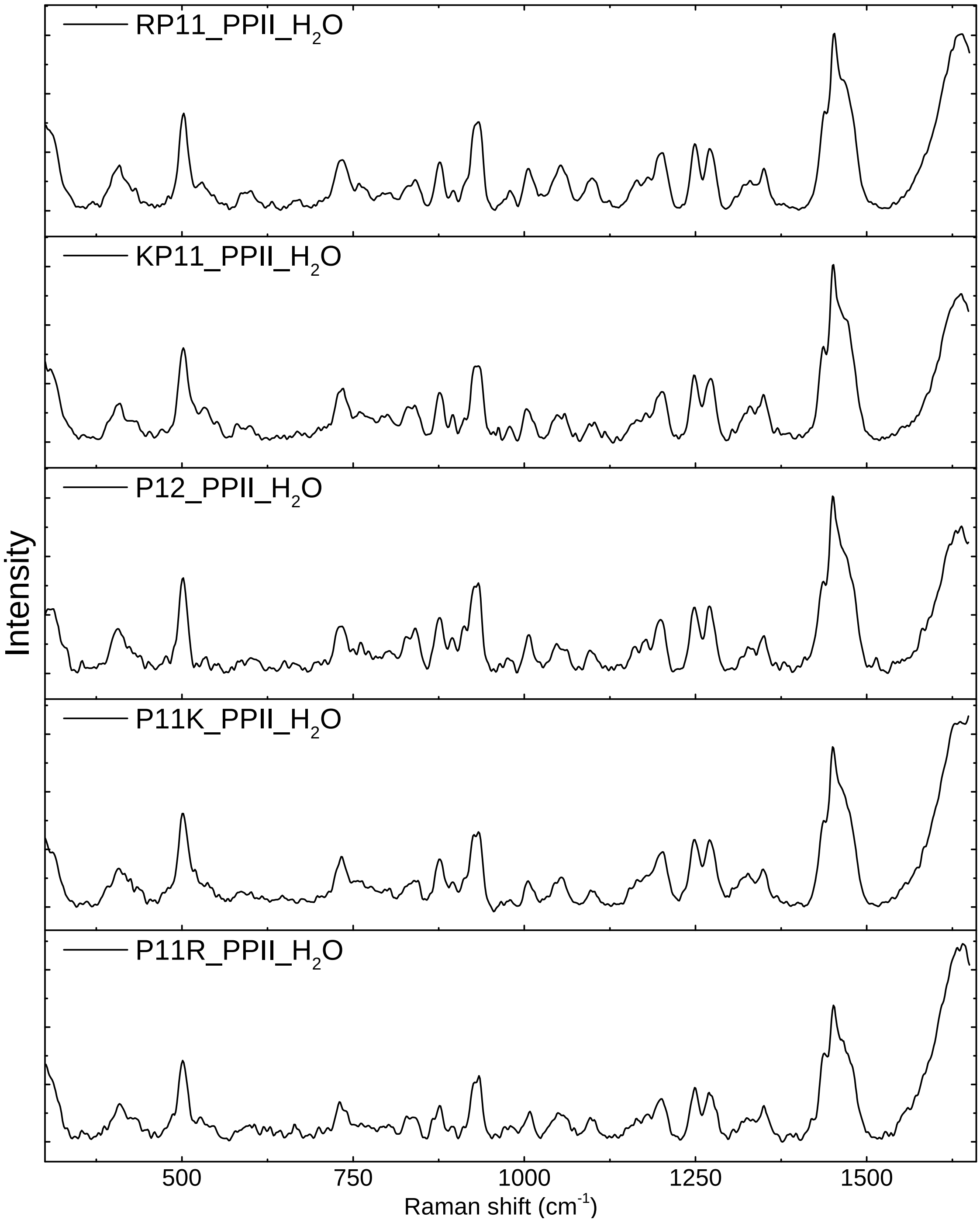

Figure S6. Raman spectra of the PPII form of P12 series in aqueous solution.

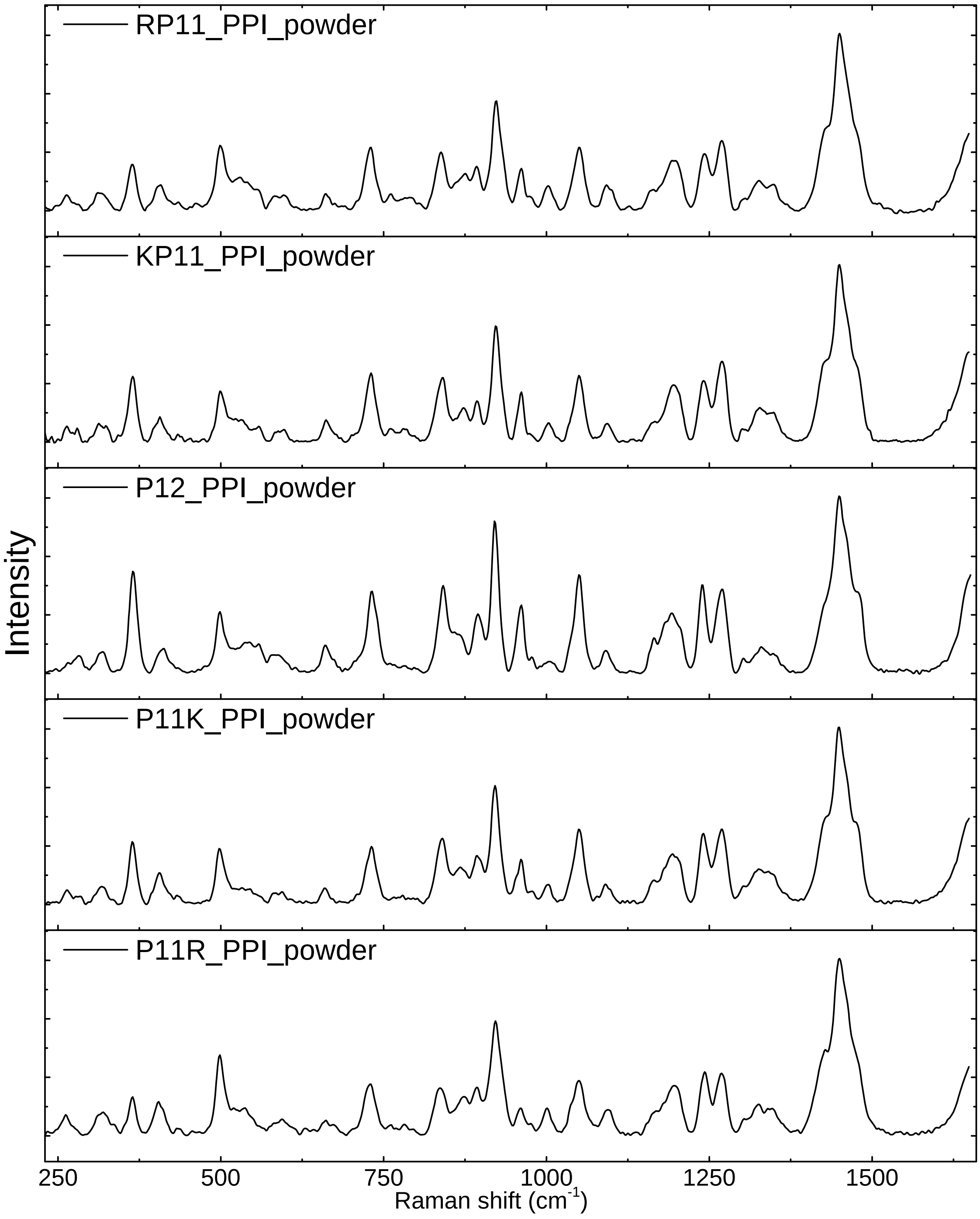

Figure S7. Raman spectra of the PPI form of P12 series powder.

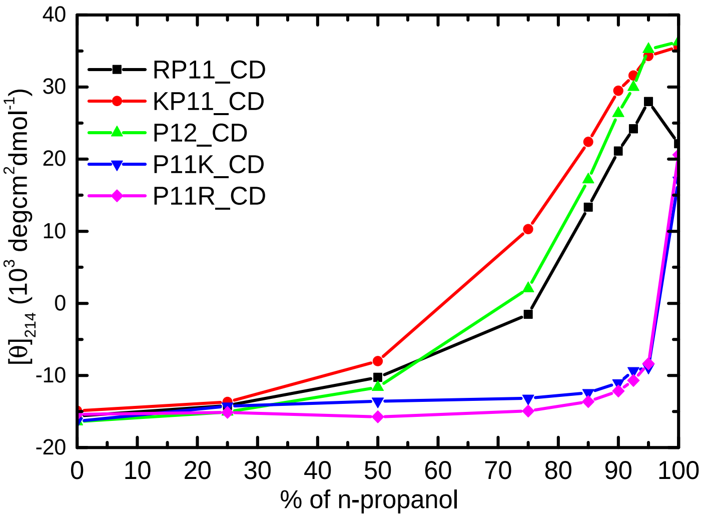

Figure S8. Plots of circular dichroism (CD) signals at 214 nm (PPI) versus the percentages of n-propanol in phosphate buffer for P12 series peptides.[27]

4. Assignment of vibrational modes to the observed Raman shifts for peptides

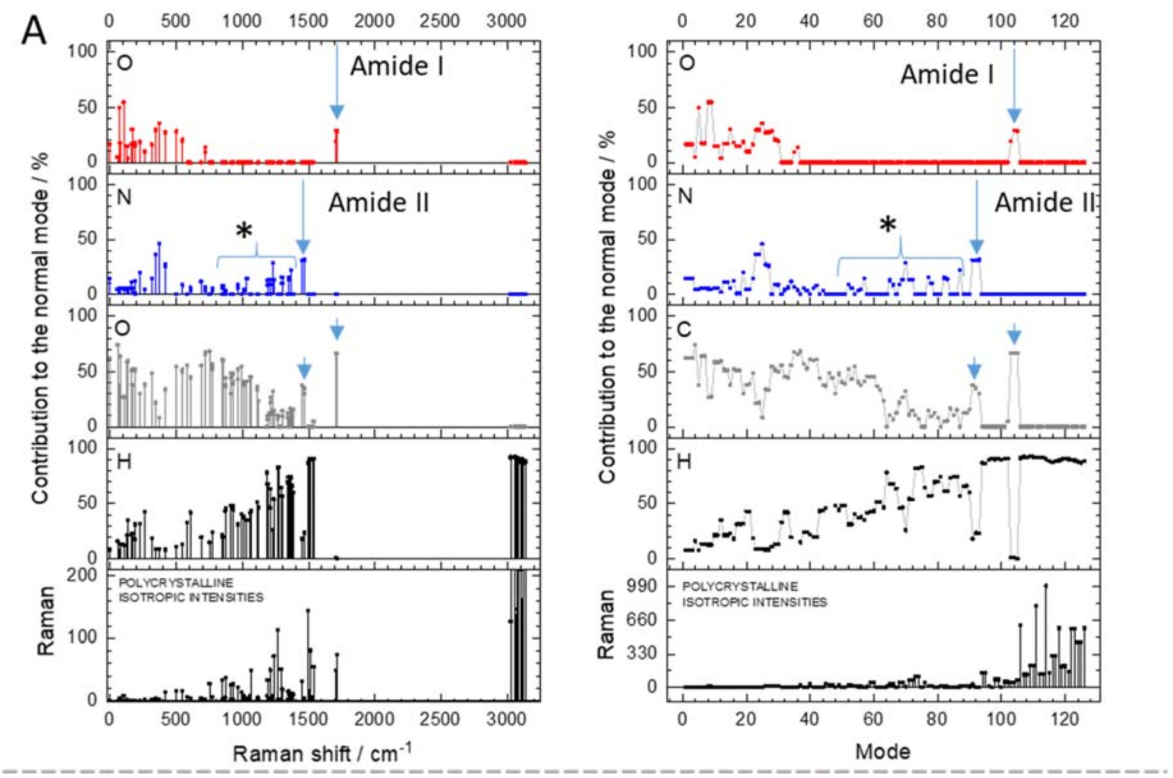

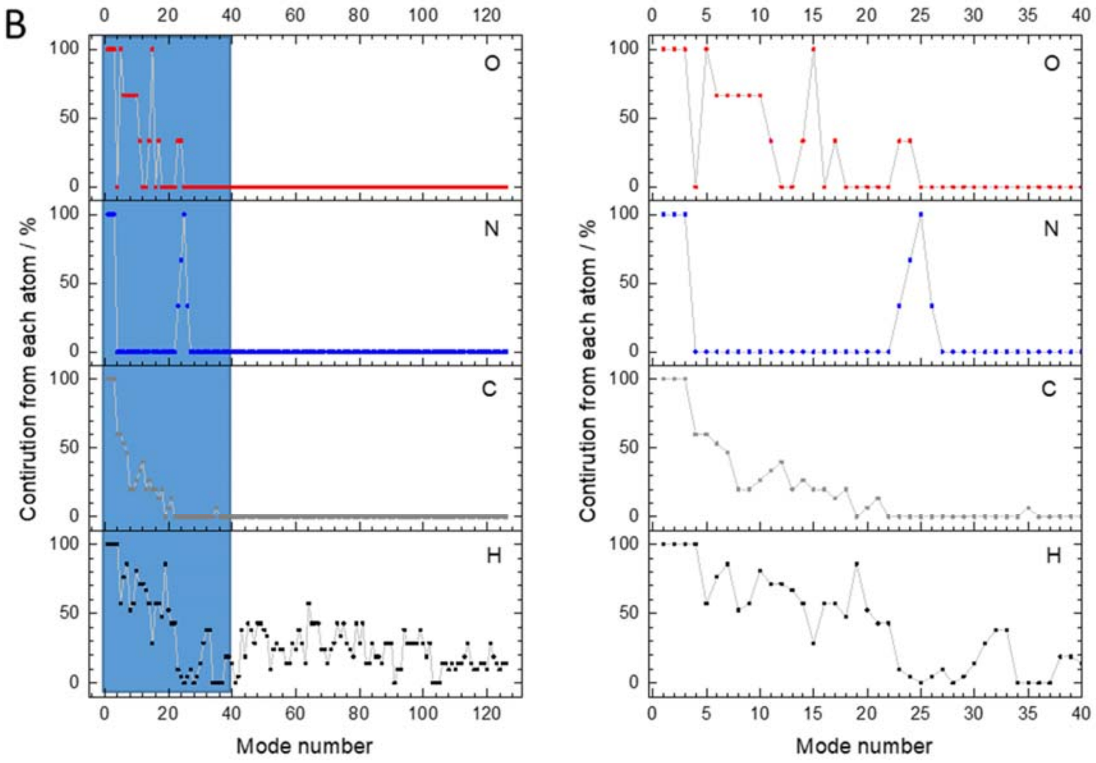

Figure S9. Mode analysis results of PPII conformation of poly-L-proline. Panels A and B show mode analyses on the amide-type motions and the collective type motions where all atoms participate in the motions, respectively. In the amide-type motions, the number of each type (O, N, C, and H) atom is counted if the displacement is larger than 0.01 Å for each direction (a, b, and c-axis). In the collective type motions, we use the total displacement larger than 0.059 Å which is determined on the basis of the three acoustic phonon modes (ω=0). In Panel A, * represents the motions due to the ring deformations.

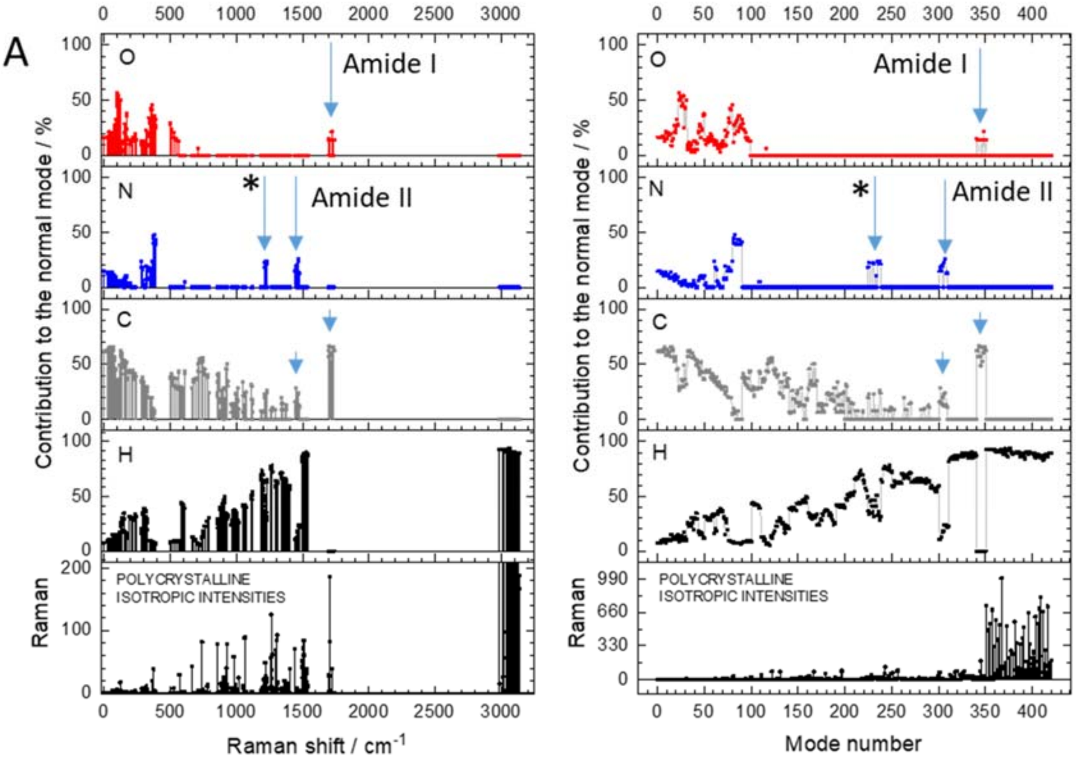
A
O
Amide I
N
*
Amide II
C
H
Contribution to the normal mode / %
Raman
POLYCRYSTALLINE
ISOTROPIC INTENSITIES
Raman shift / cm-1
Mode number

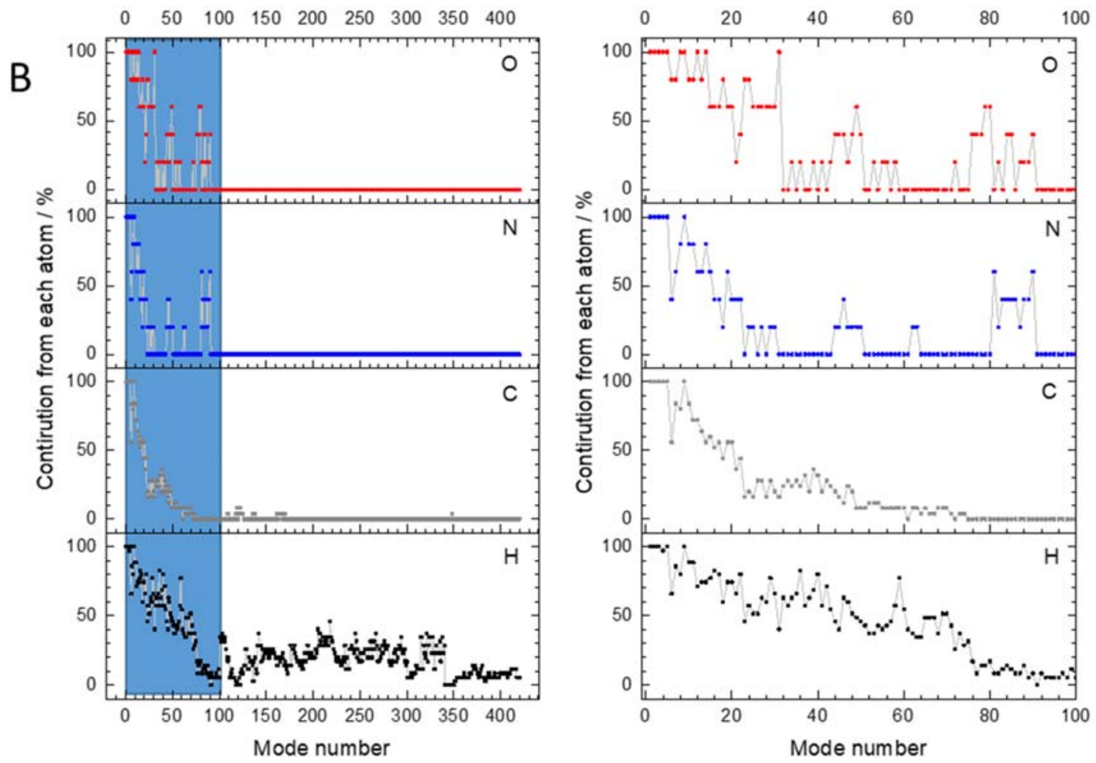

Figure S10. Mode analysis results of PPI conformation of poly-L-proline. Panels A and B show mode analysis results on the amide-type motions and the collective type motions in which all atoms participate in the motions, respectively. In the amide-type motions, the number of each type (O, N, C, and H) atom is counted if the displacement is larger than 0.01 Å for each direction (a, b, and c-axis). In the collective type motions, we use the total displacement larger than 0.032 Å which is determined on the basis of the three acoustic phonon modes (ω=0). In Panel A, * represents the motions due to the ring deformations.

3D crystal structure is quite different from 1D polymer structure as shown in Fig. S11. Both panels are views along the a-axis (1D) and c-axis (3D). Orange allow in each panel shows the distance between two carbon atoms: 1D polymer (3.067 Å) and 3D crystal (3.120 Å). This difference can be due to the existence of inter-peptide hydrogen bonding in 3D crystal case. To see this, we should note that there are two types of hydrogen bonding in 3D crystal of the polyproline II (Figure S11(b)): one acts between oxygen atom and nearest H atom (~ 2.516Å) in the same polypeptide (intra-polypeptide hydrogen bond) and the other interact with nearest H atom (~2.634 Å) of the different polypeptide (inter-polypeptide hydrogen bond). The bond distance suggests the intra-polypeptide hydrogen bonding may be stronger than hydrogen bonding acting on the different polypeptide. Polyproline I also have two types of hydrogen bonding one takes part in forming intra-polypeptide structure (2.383 Å ~ 2.561 Å) and the other one binds nearest neighboring polypeptide (~2.267 Å) (Figure S11(c)). The bond distance suggests the inter-polypeptide hydrogen bonding may be stronger than hydrogen bonding acting within the same polypeptide.

(a)

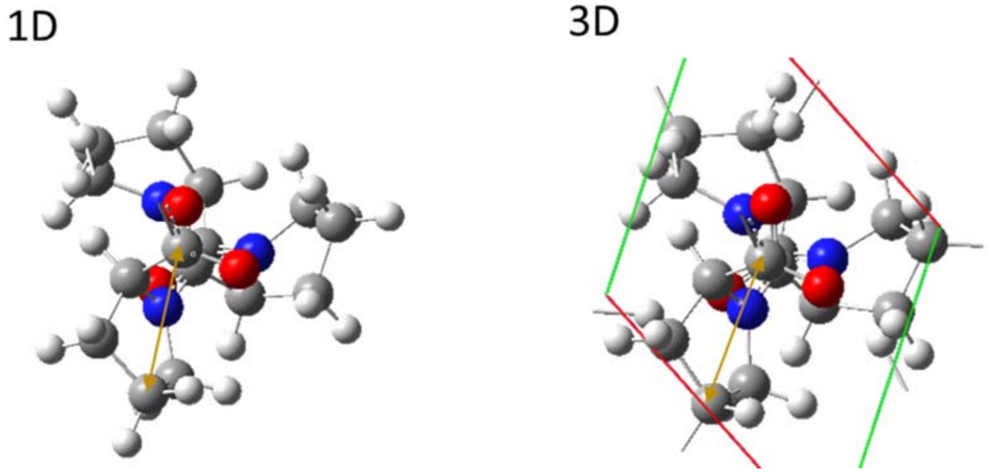

(b)

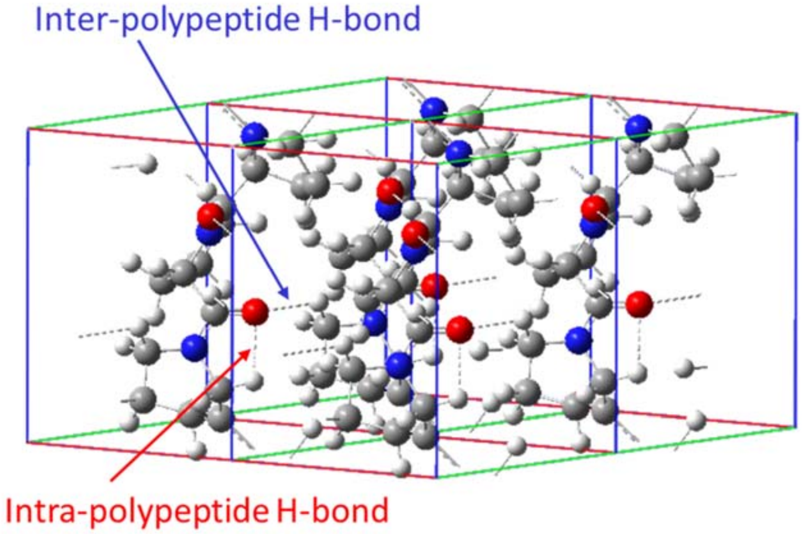

(c)

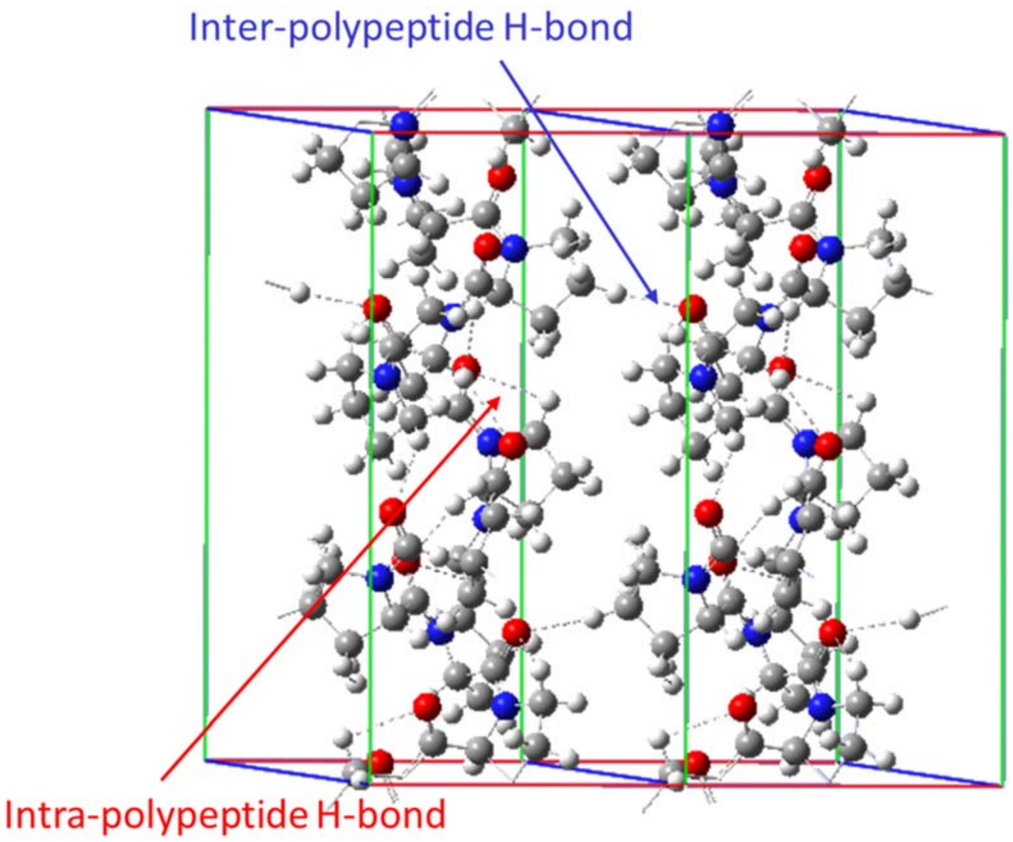

Figure S11. (a) 1D and 3D crystal structure of poly proline peptide, (b) H-bond in polyproline II, and (c) H-bond in polyproline I.

Table S1. Experimental and simulated crystal lattice of PPI and PPII conformations of poly-L-proline by using Crystal 17 at the B3LYP-D3/6-31G(d,p) level with the coupled-perturbed Kohn-Sham scheme.

	PPI		PPII	
	Expt.[a]	Calc.	Expt.[a]	Calc.
a (Å)	9.15	9.12	6.64	6.65
b (Å)	18.93	18.87	6.64	6.65
c (Å)	9.20	9.25	9.60	9.54
β (deg)	59.28	59.84	90	90
V ($Å^3$)	1369.98	1376.73 (+0.49%)	366.97	365.01 (-0.53%)

a Ref. 11.

Table S2-1. Vibrational modes of poly-L-proline II and assignments based on CRYSTAL 17 calculations. Assignments are deduced from Fig. S9.

$\omega_{expt.}$	mode #	$\omega_{calc.}$	Assignment
56	4	56	Libration around c-axis
74	6,7	* 74	spring-type motion involving pyrrolidine rings
106	8, 9	* 104	twist of carbonyl groups, twist of pyrrolidine rings
129	10,11	* 129	twist of pyrrolidine rings, twist of carbonyl groups
165	13,14	* 165	CH_2 rocking
259	20,21	* 261	CH_2 rocking
313	22	315	CH_2 rocking, NC in-plane bending (plane: pyrrolidine ring plane)
334	23,24	* 343	CH_2 rocking, NC out-of-plane bending
362	25	372	CH_2 rocking, NC out-of-plane bending, CO in-plane bending
404	26,27	* 416	NC out-of-plane bending
497	28	502	NC in-plane bending
536	29,30	* 548	NC bending, CO bending
737	37	753	CO out-of-plane bending
838	40	847	ring twist
875	45	875	ring twist
898	46,47	* 913	ring twist
920	49	931	ring deformation, C_3-C_4-C_5 stretching
1050	59,60	* 1066	ring deformation, C_3-C_4 stretching
1176	65,66	* 1191	ring deformation, CH_2 twisting, C_2-C_3 stretching
1202	71,72	* 1236	ring deformation, NC stretching (two C atoms each side of the N atom on the ring)
1242	73,74	* 1270	CH_2 twisting
1266	76	1292	ring deformation
1325	81	1346	CH_2 wagging
1343	88,89	* 1378	CH_2 wagging, ring deformation
1424	91	1451	[a]amide II
1450	94,95	* 1496	[a]CH_2 scissoring
1475	101,102	* 1537	[a]CH_2 scissoring
1650	105	1717	[a]amide I

* represents doubly degenerated state; [a] represents characteristic frequencies of the band (see Table S2-2)

Table S2-2. Characteristic bands of poly-l-proline II

**Band**	**Mode #s**	**Simulated frequencies / cm^{-1}**
CH_2 wagging	87-90	
[a]Amid II	*91(A), **92(E), **93(E)	1451.3(A), 1466.0(E)
CH_2 scissoring	94-102	1495.7-1537.1
Amide I	**103(E), **104(E), *105 (A)	1702.8(E), 1716.8(A)

[a] mixing of wagging and Amid II

* and ** represent the largest and the second largest Raman intensities in the band, respectively.

A and E represent symmetry: E is a doubly degenerated state

Note collective motions in the lower frequency region (< 500cm^{-1}) may need more detailed analysis (see Fig. S9).

Table S3-1. Vibrational mode of poly-L-proline I and their assignments based on CRYSTAL 17 calculations. Assignments are deduced from Fig. S10.

$\omega_{expt.}$	Mode #	$\omega_{calc.}$	Assignment
68	15	64	twist of pyrrolidine ring pair
75	18	76	twist of pyrrolidine ring pair, CO wagging+ spring-type motion
99	25	101	scissoring of carbonyl groups
130	31	122	CO bending from || to ⊥ helix axis, rocking of pyrrolidine rings
315	63	287	ring twist
365	72	320	ring-CO scissoring out of helix axial
410	81	374	pyrrolidine ring twist
499	95	524	NC-CO scissoring
543	100	569	NC bending, CO bending
663	111	663	ring deformation
740	122	740	CO out-of-plane bending
840	131	853	ring deformation, C_1-C_2 stretching
900	154	902	ring twist, CH_2 twisting
920	164	923	ring deformation, C_3-C_4-C_5 stretching
960	179	980	ring deformation
1050	197	1064	ring deformation, C-C asymmetric stretching
1182	228	1217	ring twist, CH_2 twisting
1239	243	1263	ring twist, CH_2 twisting
1266	257	1308	ring twist, CH_2 twisting
1327	279	1364	CH_2 wagging
1344	292	1388	CH_2 wagging
1427	301	1444	[a]amide II
1450	317	1502	[a]CH_2 scissoring
1479	336	1530	[a]CH_2 scissoring
1650	345	1708	[a]amide I

[a] represents characteristic frequencies of the band (see Table S3-2)

Table S3-2 Characteristic bands of poly-l-proline I

**Band**	**Mode #s**	**Simulated frequencies / cm^{-1}**
Amide II+ CH_2 scissoring	303-310	1455.2~1475.0
CH_2 scissoring	311-340	1494.3~1536.9
Amide I	341,342,343, **344, *345, 346,347,348,349,350	1698.1,1698.5,1702.1, **1703.9, **1707.9,1709.0,1717.5,1720.3, 1728.9,1736.2

* and ** represent the largest and the second largest Raman intensities in the band, respectively.
Note collective motions in the lower frequency region (< 500cm^{-1}) may need more detailed analysis (see Fig. S10).

Table S4. Vibrational mode of the PPII form of P12 and their assignments based on Gaussian 09 calculations.

$\omega_{expt.}$	$\omega_{calc.}$	Assignment
261	266	CH_2 rocking
310	321	CH_2 rocking, NC out-of-plane bending (plane: pyrrolidine ring plane)
334	354	CH_2 rocking, NC out-of-plane bending, CO in-plane bending
404	409	NC out-of-plane bending
499	503	NC in-plane bending
529	537	NC bending, CO bending
730	747	CO out-of-plane bending
762	788	CH_2 twist
833	848	ring deformation
875	898	ring twist
898	920	ring twist
925	937	ring twist
1050	1085	ring deformation, C-C stretching
1098	1132	ring twist
1168	1185	ring deformation, CH_2 twist
1177	1211	CH_2 twisting
1203	1228	CH_2 wagging
1248	1285	CH_2 twisting
1270	1313	CH_2 twisting
1328	1381	CH_2 wagging
1350	1393	CH_2 wagging
1424	1460	amide II
1454	1531	CH_2 scissoring
1482	1550	CH_2 scissoring
1650	1643	amide I

Table S5. Vibrational mode of the PPI form of P12 and their assignments based on Gaussian 09 calculations.

$\omega_{expt.}$	$\omega_{calc.}$	Assignment
320	332	CH_2 wagging
365	361	ring-CO scissoring (CO bending out of helix axis)
412	395	pyrrolidine ring twist
499	513	CH_2 rocking
583	603	CH_2 rocking
660	642	CO bending of terminal COO^-, ring twist
731	719	CO out-of-plane bending
842	802	ring twist
865	839	ring twist
895	895	ring twist, CH_2 rocking
920	940	ring deformation
960	977	ring deformation
1050	1071	ring deformation, C-C asymmetric stretching
1092	1130	ring twist
1165	1193	ring deformation, CH_2 wagging
1182	1214	CH_2 twisting
1193	1239	CH_2 wagging
1240	1284	CH_2 twisting
1270	1335	CH_2 wagging
1302	1356	CH_2 wagging
1330	1375	CH_2 wagging
1350	1388	CH_2 wagging
1425	1450	Am II
1449	1533	CH_2 scissoring
1479	1546	CH_2 scissoring
1650	1625	Am I
